# Clinical characteristics, tongue manifestations, and traditional Chinese medicine syndrome patterns of spring influenza A in children: a single-center retrospective study

**DOI:** 10.3389/fped.2026.1879124

**Published:** 2026-07-16

**Authors:** Yuan Si, Xiang Zhao

**Affiliations:** 1 Department of Traditional Chinese Medicine, People’s Hospital Affiliated to Shandong First Medical University, Jinan, China; 2 Immunization Planning Division, Qingdao Shibei District Center for Disease Control and Prevention, Qingdao, China

**Keywords:** children, clinical characteristics, influenza a, TCM syndrome differentiation, tongue manifestations

## Abstract

**Objective:**

To systematically analyze the clinical manifestations, dynamic changes in hematological parameters, traditional Chinese medicine (TCM) tongue characteristics, and syndrome differentiation patterns in children with influenza A during spring, so as to provide clinical evidence for the integrated Chinese and Western medicine diagnosis and treatment of pediatric influenza A in spring.

**Methods:**

A single-center retrospective study design was adopted. A total of 143 children who visited the Department of Traditional Chinese Medicine of our hospital in the spring of 2024 and 2025 and were initially diagnosed with influenza A were included. General information, initial symptoms, and symptom combinations were collected. Hematological parameters were measured, including white blood cell count (WBC), absolute neutrophil count (NEU), absolute lymphocyte count (LYM), neutrophil-to-lymphocyte ratio (NLR), procalc itonin (PCT), and C-reactive protein (CRP). According to disease duration, patients were divided into three stages: <1 d, >1–≤3 d, and >3–14 d, and trends of change were compared. Tongue characteristics including tongue color, coating texture, and coating color were recorded, and TCM syndrome differentiation types were statistically analyzed.

**Results:**

(1) General data and symptom characteristics: Among the 143 children, 78 were male (54.55%) and 65 were female (45.45%), with a mean age of (6.48 ± 3.61) years. The main age group was >5–≤10 years (64 cases, 44.76%). The mean disease duration was (1.82 ± 1.69) d, and 100 cases (69.93%) sought medical care within ≤1 d. Fever was the most common initial symptom (132 cases, 92.31%). The most frequent symptom combination was “ fever + cough” (116 cases, 81.12%), followed by “fever + constipation” (85 cases, 59.44%). (2) Changes in hematological parameters: At the initial visit, 129 cases (90.21%) had LYM below the normal reference range, and 84 cases (58.74%) had elevated PCT. With disease progression, WBC showed a s light downward trend without statistical significance (*P* > 0.05); NEU gradually decreased, and was lower in the >3–14 d stage than in the <1 d stage (*P* < 0.05); LYM gradually increased, and was higher in the >1–≤3 d and >3–14 d stages than in the <1 d stage (*P* < 0.05); NLR gradually decreased, and was lower in the >3–14 d stage than in the <1 d stage (*P* < 0.05). PCT and CRP increased in the >1–≤3 d stage and decreased in the >3–14 d stage; PCT in the >3–14 d stage was lower than that in the >1–≤3 d stage (*P* < 0.05), and CRP in the >1–≤3 d stage was higher than that in the <1 d stage (*P* < 0.05). (3) Tongue characteristics: Red tongue was predominant (134 cases, 93.71%), and the proportion of red tongue increased with disease progression (<1 d 92.00%, >3–14 d 100%). Thick coating was predominant (91 cases, 63.64%), and the proportion increased with prolonged disease duration (<1 d 60.00%, >3–14 d 73.33%). White coating was the most common (89 cases, 62.24%), while the proportion of yellow coating increased with disease progression (<1 d 30.00%, >3– 14 d 60.00%). (4) TCM syndrome differentiation characteristics: The distribution of syndromes was as follows: heat-toxin attacking the lung syndrome (50 cases, 34.97%), wind-heat invading the exterior syndrome (47 cases, 32.87%), wind-cold constraining the exterior syndrome (29 cases, 20.28%), and dampness obstructing the defensive qi syndrome (17 cases, 11.89%). Stratification by sex showed that wind-heat invading the exterior syndrome was slightly more common in males (35.90%), while heat-toxin attacking the lung syndrome was slightly more common in females (38.46%). Stratification by age showed that in the 0–≤5 years and >5–≤10 years groups, wind-heat invading the exterior syndrome was more common (35.71% and 35.94%, respectively), whereas in the >10–16 years group, heat-toxin attacking the lung syndrome predominated (56.52%). Stratification by disease duration showed that the >3–14 d stage had the highest proportion of heat-toxin attacking the lung syndrome (53.33%).

**Conclusion:**

Spring influenza A in children was characterized by fever-dominant onset, frequent cough and constipation, early lymphopenia, dynamic inflammatory marker changes, and progressive tongue/coating changes across disease-duration stages. The observed increase in red tongue, thick coating, yellow coating, and heat-related TCM syndromes suggests a possible disease-duration-related shift toward intensified heat signs. These findings may provide exploratory reference for integrated clinical assessment, but they should be interpreted with caution because of the single-center retrospective design, small late-stage subgroup, absence of treatment/outcome data, and lack of standardized digital tongue imaging. Prospective multicenter studies are needed to validate these observations and clarify their clinical value.

## 1 Introduction

Influenza A is an acute respiratory infectious disease caused by influenza A virus, characterized by high infectiv ity and obvious seasonal epidemic features (1). Due to immature immune function, frequent group contact, and relatively weak respiratory barrier function, children constitute an important susceptible population during influenza epidemics (2). In spring, the climate transitions from cold to warm, with frequent temperature fluctuations and active wind pathogens, increasing the opportunity for exogenous pathogens to invade the body. Children are therefore more prone to onset during this period (3). Thus, exploring the clinical characteristics of influenza A in children during spring has important practical significance. Modern medicine holds that influenza A usually presents with acute onset, mainly manifested by fever, cough, sore throat, and fatigue, and may be accompanied by varying degrees of systemic inflammatory response and respiratory mucosal injury (4, 5). Due to considerable individual differences, pediatric patients exhibit variable clinical severity; in some cases, the disease may progress rapidly and even be complicated by lower respiratory tract infections such as pneumonia (6). In recent years, research on pediatric influenza has mainly focused on vi rological characteristics, epidemic trends, and antiviral therapy (7, 8), whereas systematic analyses of clinical manifestations and dynamic changes in inflammatory responses remain to be further improved. Particularly at different stages of disease duration, the intrinsic patterns of disease evolution and their relationship with clinical manifestations have not yet been systematically clarified.

From the perspective of traditional Chinese medicine (TCM), influenza A generally falls into the categories of “seasonal common cold”, “warm disease”, or “epidemic disease”. Its onset is mostly attributed to the invasion of external wind pathogens or warm-heat pathogens affecting the lung and defensive systems, resulting in an imbalance between vital qi and pathogenic factors (9). Children are physiologically characterized by “delicate zang-fu organs”, “insufficient form and qi”, and a “pure yang constitution”. Therefore, after invasion by external pathogens, children are more likely to experience rapid heat transformation and dynamic syndrome changes (10). To avoid repetitive theoretical description, the present study used the classical TCM concept of exterior-to-interior progression mainly as an interpretive framework for analyzing whether clinical symptoms, tongue manifestations, and syndrome patterns vary across different stages of disease duration. In spring, wind-heat and dampness may interact with the lung and spleen systems, leading to fever, cough, poor appetite, constipation, and other manifestations (11).

Tongue manifestation is an important basis for TCM syndrome differentiation and treatment, reflecting the status of qi and blood, the waxing and waning of pathogenic and vital factors, and the depth of disease location. Changes in tongue color indicate cold-heat properties and blood-level conditions; the thickness of tongue coating reflects the strength of pathogenic factors and disease severity; and changes in coating color are closely related to disease evolution (12, 13). In recent years, pediatric TCM and integrated Chinese and Western medicine studies on influenza have gradually increased. A multicenter randomized clinical trial showed that Qinxiang Qingjie oral solution had comparable efficacy to oseltamivir in children with influenza and TCM-defined exterior-interior heat syndromes (14). Another randomized, double-blinded, parallel-controlled clinical trial protocol was designed to evaluate Reduning injection for pediatric influenza compared with oseltamivir (15). More recently, a multicenter randomized non-inferiority trial reported that Xiao'er Fengre Qing oral liquid was non-inferior to oseltamivir in treating pediatric influenza with wind-heat invading the defense syndrome (16). These studies suggest that pediatric influenza has become an important area of clinical research in TCM and integrated Chinese and Western medicine.

However, most existing pediatric studies have focused on treatment efficacy, medication evaluation, or trial protocols, whereas real-world evidence linking disease duration, hematological dynamics, tongue manifestations, and TCM syndrome distribution remains limited. Furthermore, the disease course of pediatric influenza changes rapidly, and clinical syndromes may evolve over time. Failure to identify changes in pathogenesis in a timely manner may affect the accuracy of syndrome differentiation and clinical assessment. Therefore, based on clinical practice, systematic analysis of clinical manifestations, syndrome distribution patterns, and tongue changes in children with influenza A during spring may deepen understanding of disease evolution and provide more objective references for integrated Chinese and Western medicine assessment. Based on the above background, this single-center retrospective study included children diagnosed with influenza A during spring. Through retrospective analysis of clinical data and TCM syndrome differentiation information, this study systematically explored age characteristics of onset, symptom presentation patterns, tongue manifestations, and syndrome distribution features, and further analyzed disease evolution trends at different stages of disease duration. The aim was to provide exploratory clinical evidence for the integrated Chinese and Western medicine assessment of pediatric influenza A in spring and to offer a basis for subsequent prospective studies.

## 2 Materials and methods

### 2.1 Study design and ethical statement

This study adopted a single-center, retrospective observational design. Children who visited the outpatient Department of Traditional Chinese Medicine of our hospital during the spring seasons of 2024 and 2025 and were diagnosed with influenza A were consecutively screened from the hospital information system and laboratory information system according to predefined inclusion and exclusion criteria. The study data were derived from previous outpatient medical records and laboratory reports. Data collected included demographic information, first-visit symptoms, disease duration, hematological parameters, TCM tongue manifestations, and TCM syndrome differentiation records. Cases with missing key variables were excluded.

**Figure 1:**
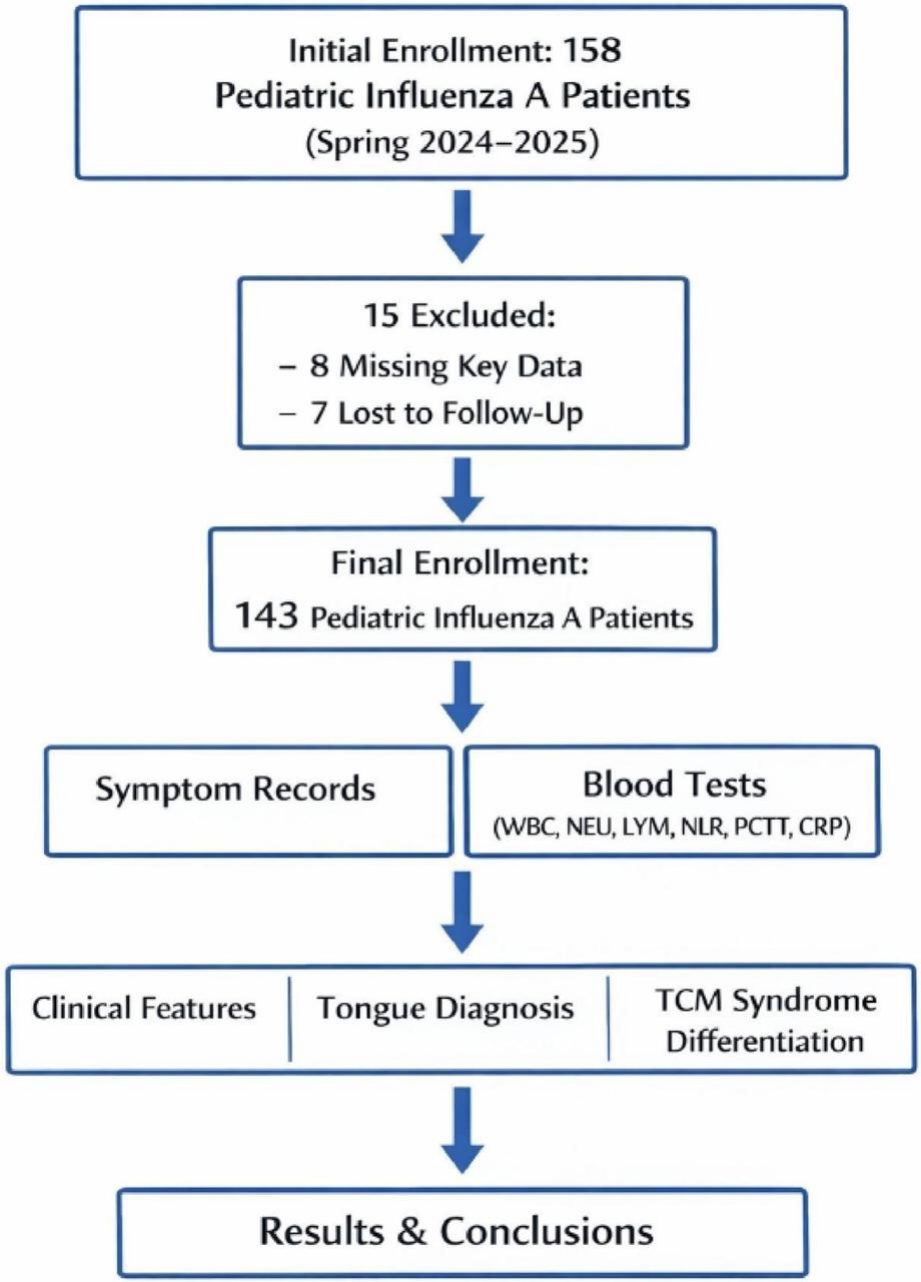
Flowchart of participant enrollment and study design of this study.

This study focused on the clinical characteristics, hematological parameters, tongue manifestations, and TCM syndrome distribution of children with influenza A at different disease-duration stages. Descriptive and comparative analyses were performed to explore the relationship between disease duration and relevant clinical or TCM indicators. Treatment regimens after the first visit and prognosis-related outcomes were not included as primary analytical variables. The flowchart of participant enrollment and study design is shown in Figure 1.

This retrospective study did not involve additional interventions. During data analysis, patient information was anonymized, and patient privacy was strictly protected. The study protocol was submitted to and approved by the Ethics Committee of our hospital (Ethics approval number: LG24-ZYLC08), in compliance with relevant medical ethics requirements.

### 2.2 Study subjects

The cases were derived from children who visited the outpatient Department of Traditional Chinese Medicine of our hospital in the spring of 2024 and 2025. The hospital information system (HIS) and laboratory information system (LIS) were searched using the terms “influenza A/flu A/Influenza A” and related diagnostic and laboratory records. Eligible cases were screened in combination with outpatient medical records. All included cases were first-visit patients, defined as those who sought medical attention for the first time due to the current episode of acute upper respiratory tract infection – related symptoms and completed diagnostic evaluation. The Western medicine diagnostic criteria referred to the Expert Consensus on the Diagnosis and Treatment of Influenza in Children (2020 Edition) (17); the TCM diagnostic criteria referred to the Guidelines for Integrated Traditional Chinese and Western Medicine Diagnosis and Treatment of Pediatric Influenza (18). (1) Inclusion criteria:

① age 0–16 years, no restriction on sex; ② visit during the spring season of the study year; ③ meeting the clinical manifestations of influenza A and confirmed as influenza A infection by etio logical testing; ④ complete data, including general information and main symptom records, with tongue manifestation description or tongue image/outpatient record available for interpretation, and key laboratory or inflammation-related test records; ⑤ first visit to the Department of Traditional Chinese Medicine of our hospital during the current disease episode. (2) Exclusion criteria: ① coexisting confirmed severe bacterial infection or other definite pathogen infection (such as adenovirus, Mycoplasma infection, etc.) as the primary cause; ② previous underlying diseases affecting immune/inflammatory responses, such as congenital immunodeficiency, malignant tumors, long-term use of glucocorticoids or immunosuppressants; ③ previous significant chronic respiratory diseases in acute exacerbation stage (such as acute attack of moderate to severe asthma, recurrent infection of bronchiectasis, etc.); ④ severe missing data, with key variables (disease duration, main symptoms, syndrome differentiation type, or tongue manifestation elements) missing and unable to be supplemented.

### 2.3 Grouping and disease duration stratification

#### 2.3.1 Definition of disease duration

Disease duration was defined from the onset time of initial symptoms (as described by the guardian and recorded in the medical record, usually based on the first occurrence of fever or respiratory symptoms) to the day of visit, and calculated as disease duration in days (d).

#### 2.3.2 Stratification of disease duration

According to the clinical evolution pattern of influenza A and outpatient visit characteristics, disease duration was divided into: (1) ≤1 d; (2) >1–≤3 d; (3) >3–14 d. This stratification was used to describe differences in clinical manifestations, tongue features, and syndrome distribution among children at different stages, as well as to analyze trends of related laboratory indicators during the course of disease.

#### 2.3.3 Age stratification

According to children 's growth and developmental characteristics and clinical staging, age was divided into: (1) 0–≤5 years; (2) >5–≤10 years; (3) >10–16 years. This was used to compare differences in symptom spectrum characteristics and syndrome distribution among different age groups.

### 2.4 Data collection content and methods

#### 2.4.1 General information and physical sign data

Information collected included sex, age, date of visit, time of initial symptom onset, disease duration at visit (d), past medical history and allergy history (as recorded in medical records), and body temperature at visit. Main symptoms were extracted from outpatient first-visit medical records, including but not limited to: fever, cough, sore throat, nasal congestion and r hinorrhea, fatigue, headache, myalgia, nausea and vomiting, abdominal pain, diarrhea, constipation, etc. To reduce information bias, symptom recording followed these principles: (1) based on chief complaint and history of present illness clearly documented in the first-visit record; (2) new symptoms appearing at revisit were only used for describing “disease course evolution” and were not included in first-visit symptom spectrum statistics; (3) symptom combinations were organized to analyze common co-occurrence patterns.

#### 2.4.2 Laboratory data

Posterior pharyngeal wall and nasal swab specimens were collected from children and preserved in viral transport medium for testing. After culture, reverse transcription—polymerase chain reaction (RT-PCR) was used to detect influenza A virus, influenza B virus, etc. (kits purchased from Wuhan Z hongzhi Biotechnology Co., Ltd.). Peripheral blood samples were collected for routine blood tests and inflammatory indicators, including: white blood cell count (WBC), absolute neutrophil count (NEU), absolute lymphocyte count (LYM), neutrophil-to-lymphocyte ratio (NLR), procalc itonin (PCT), and C-reactive protein (CRP). The normal reference ranges were based on the health industry standard WS/T779-2021 “ Reference Intervals for Blood Cell Analysis in Children” (19).

#### 2.4.3 Collection and interpretation of TCM tongue manifestations

Tongue information was derived from tongue descriptions in outpatient medical records and/or tongue photographs. Tongue interpretation followed standardized TCM tongue diagnosis criteria (20). Recorded elements included tongue color, coating texture, and coating color. Tongue color was classified as pale red, red, or dark purple; coating texture was classified as thin, thick, greasy, or peeled according to coating thickness and tongue body exposure; and coating color was classified as white or yellow. Because this was a retrospective study, tongue photographs were obtained during routine outpatient care rather than under a standardized digital acquisition protocol. Therefore, quantitative colorimetric or texture-based image analysis was not performed. To improve consistency, two physicians with clinical experience in TCM pediatrics or TCM internal medicine independently assessed tongue findings using the same categorical criteria. Records or photographs with unclear tongue information were excluded. During interpretation, age-related physiological variations were considered together with clinical context. In younger children, a relatively pale-red or tender tongue body and thin white coating were not directly regarded as pathological findings unless accompanied by corresponding clinical manifestations, disease-duration changes, or abnormal hematological/inflammatory indicators. In contrast, markedly red or dark purple tongue, thick or greasy coating, and yellow coating were interpreted as disease-associated findings when supported by fever intensity, respiratory or gastrointestinal symptoms, disease duration, and laboratory changes. Before consensus discussion, inter-rater agreement between the two physicians was evaluated using Cohen's kappa coefficient. The kappa values for tongue color, coating texture, and coating color were 0.84, 0.78, and 0.81, respectively, indicating substantial agreement. Disagreements were resolved through discussion, and the final consensus results were used for statistical analysis.

#### 2.4.4 TCM syndrome differentiation criteria and recording

Syndrome differentiation was based on TCM pediatric exogenous febrile disease and warm disease theories, with reference to commonly used clinical differentiation standards (21). According to symptoms, tongue manifestations, and available clinical records at the first visit, comprehensive judgment was made. The main syndrome types included wind-cold constraining the exterior syndrome, wind-heat invading the exterior syndrome, dampness obstructing the defensive qi syndrome, and heat-toxin attacking the lung syndrome.

Operational criteria were defined as follows. Wind-cold constraining the exterior syndrome was characterized mainly by acute onset with fever, aversion to cold or wind, nasal congestion or clear rhinorrhea, cough, absence of obvious thirst, and a pale-red tongue or thin white coating. Wind-heat invading the exterior syndrome was characterized mainly by fever, mild aversion to wind, cough, sore throat, rhinorrhea or turbid nasal discharge, thirst, red or pale-red tongue, and thin white or thin yellow coating. Dampness obstructing the defensive qi syndrome was characterized mainly by fever with heaviness or fatigue, poor appetite, nausea or abdominal discomfort, loose stool or sticky stool tendency, chest or epigastric oppression, and thick or greasy tongue coating, usually white or slightly yellow. Heat-toxin attacking the lung syndrome was characterized mainly by persistent or high fever, obvious cough, sore throat, yellow sputum or thick respiratory secretions, thirst, constipation, red tongue, and yellow or thick coating; elevated inflammatory indicators were used as ancillary evidence when available.

For each case, the recorded syndrome type was based on the syndrome explicitly documented by the attending physician at the first visit. If the syndrome type was not explicitly stated but complete information from the four diagnostic methods was available, supplementary differentiation was conducted by the research team according to the above unified criteria. In general, classification required the presence of the dominant clinical pattern together with compatible tongue findings. The supplementary process also adopted a two-person independent assessment and consensus discussion method to improve consistency and reproducibility.

### 2.5 Statistical methods

GraphPad Prism 8 was used for data visualization and statistical analysis. Normality of continuous variables was assessed using the Shapiro–Wilk test. The main continuous variables included in disease-duration comparisons did not show significant deviation from normality based on the Shapiro–Wilk test and distribution inspection; therefore, measurement data were expressed as mean ± standard deviation. Comparisons between predefined disease-duration groups were performed using independent-samples *t*-tests, and Bonferroni correction was applied for multiple pairwise comparisons. Categorical data were expressed as number and percentage and were compared using the *χ*^2^ test or Fisher's exact test, as appropriate. Cohen's kappa coefficient was used to assess inter-rater reliability for tongue manifestation interpretation before consensus discussion. No formal *a priori* sample size calculation was performed because of the retrospective observational design. The sample size was determined by the number of eligible children with complete clinical, laboratory, and TCM tongue/syndrome information during the predefined study period. Therefore, the findings should be interpreted as exploratory, especially for subgroup comparisons involving the >3–14 d stage. A two-sided adjusted *P* value <0.05 was considered statistically significant.

## 3 Results

### 3.1 Baseline data of children with influenza A

A total of 143 children with influenza A were included in this study. There were 78 males (54.55%) and 65 females (45.45%), with a roughly comparable sex ratio. The mean age was (6.48 ± 3.61) years. Children aged >5–≤10 years accounted for 64 cases (44.76%) and constituted the main age group of onset; 56 cases (39.16%) were aged 0–≤5 years, and 23 cases (16.08%) were aged >10–16 years. The mean disease duration was (1.82 ± 1.69) d. Among them, 100 cases (69.93%) visited within ≤1 d, 28 cases (19.58%) within >1–≤3 d, 14 cases (9.79%) within >3–≤7 d, and 1 case (0.70%) within >7–14 d, indicating that most children sought medical care at an early stage of disease onset. See Table 1.

**Table 1:** Baseline data of children with influenza A.

Item	No. of cases (*n* = 143)	Mean
Sex	–	–
Male	78 (54.55%)	–
Female	65 (45.45%)	–
Age (years)	–	–
0–≤5	56 (39.16%)	
>5–≤10	64 (44.76%)	6.48 ± 3.61
>10–16	23 (16.08%)	
Disease duration (d)	–	–
≤1	100 (69.93%)	
>1∼≤3	28 (19.58%)	1.82 ± 1.69
>3∼≤7	14 (9.79%)	
>7∼14	1 (0.70%)	

### 3.2 Analysis of symptom characteristics in children with influenza A

#### 3.2.1 Initial symptom profile

Among the 143 children, fever was the predominant initial symptom. A total of 132 cases (92.31%) presented with fever as the initial symptom, which was markedly higher than other symptoms; cough was reported in 9 cases (6.29%); rhinorrhea and sore throat were each reported in 1 case (0.70%). These results suggest that fever is the primary initial manifestation of influenza A in children during spring.

#### 3.2.2 Symptom distribution

Overall, systemic and respiratory symptoms predominated in children with influenza A, and some children also had gastrointestinal symptoms. Systemic symptoms had the highest incidence, occurring in 141 cases (98.60%), including fever in 141 cases (98.60%). Fever severity was mainly moderate and high. Respiratory symptoms occurred in 133 cases (93.01%), with cough being the most common (116 cases, 81.12%). Gastrointestinal symptoms occurred in 106 cases (74.13%), with constipation having the highest incidence (87 cases, 60.84%). See Table 2.

**Table 2:** Symptom distribution in children with influenza A.

Symptom	No. of cases (*n* = 143)	Total
Systemic symptoms	–	141 (98.60%)
Fever	141 (98.60%)	–
Low	15 (10.49%)	–
Moderate	67 (46.85%)	–
High	59 (41.26%)	–
Headache	8 (5.59%)	–
Myalgia	4 (2.80%)	–
Fatigue	2 (1.40%)	–
Respiratory symptoms	–	133 (93.01)
Cough	116 (81.12%)	–
Sputum production	43 (30.07%)	–
Sore throat	56 (39.16%)	–
Throat itching	6 (4.20%)	–
Hoarseness	4 (2.80%)	–
Nasal congestion	4 (2.80%)	–
Rhinorrhea	56 (39.16%)	–
Gastrointestinal symptoms	–	106 (74.13%)
Abdominal pain	30 (20.98%)	–
Nausea	5 (3.50%)	–
Vomiting	15 (10.49%)	–
Loose stools	9 (6.29%)	–
Constipation	87 (60.84%)	–

#### 3.2.3 Ranking of symptom combinations

The frequencies of individual symptoms from high to low were: fever (141 cases), cough (116 cases), constipation (87 cases), sore throat (56 cases), rhinorrhea (56 cases), sputum production (43 cases), abdominal pain (30 cases), vomiting (15 cases), loose stools (9 cases), headache (8 cases), throat itching (6 cases), nausea (5 cases), myalgia (4 cases), hoarseness (4 cases), nasal congestion (4 cases), and fatigue (2 cases). Analysis of symptom combinations showed that “fever + cough” was the most common (116 cases, 81.12%); followed by “fever + constipation” (85 cases, 59.44%); “fever + sore throat” and “fever + rhinorrhea” were each present in 56 cases (39.16%); “fever + sputum production” in 43 cases (30.07%); and “fever + abdominal pain” in 30 cases (20.98%). The remaining symptom combinations had relatively low incidence. See Table 3.

**Table 3:** Ranking of symptom combinations in children with influenza A.

Symptom combination	No. of cases (*n* = 143)
Fever + cough	116 (81.12%)
Fever + constipation	85 (59.44%)
Fever + sore throat	56 (39.16%)
Fever + rhinorrhea	56 (39.16%)
Fever + sputum production	43 (30.07%)
Fever + abdominal pain	30 (20.98%)
Fever + vomiting	15 (10.49%)
Fever + loose stools	9 (6.29%)
Fever + headache	8 (5.59%)

### 3.3 Comparison of hematological test indices

#### 3.3.1 Blood test results at the initial visit in children with influenza A

At the initial visit, WBC, NEU, NLR, and CRP were mostly within the normal reference ranges. A marked decrease in LYM was observed: 129 cases (90.21%) were below the normal reference range. Elevated PCT was found in 84 cases (58.74%). See Table 4.

**Table 4:** Blood test results at the initial visit in children with influenza A.

Item	Test value	Below reference	Above reference
WBC (×10^9^/L)	6.32 ± 2.58	38 (26.57%)	6 (4.20%)
NEU (×10^9^/L)	4.15 ± 2.31	3 (2.10%)	17 (11.89%)
LYM (×10^9^/L)	1.48 ± 0.82	129 (90.21%)	0 (0%)
NLR	3.72 ± 2.94	14 (9.79%)	45 (31.47%)
PCT (ng/mL)	0.16 ± 0.15	0 (0%)	84 (58.74%)
CRP (mg/L)	5.42 ± 6.21	0 (0%)	22 (15.38%)

#### 3.3.2 Changes in blood test indices over the disease course in children with influenza A

**Figure 2:**
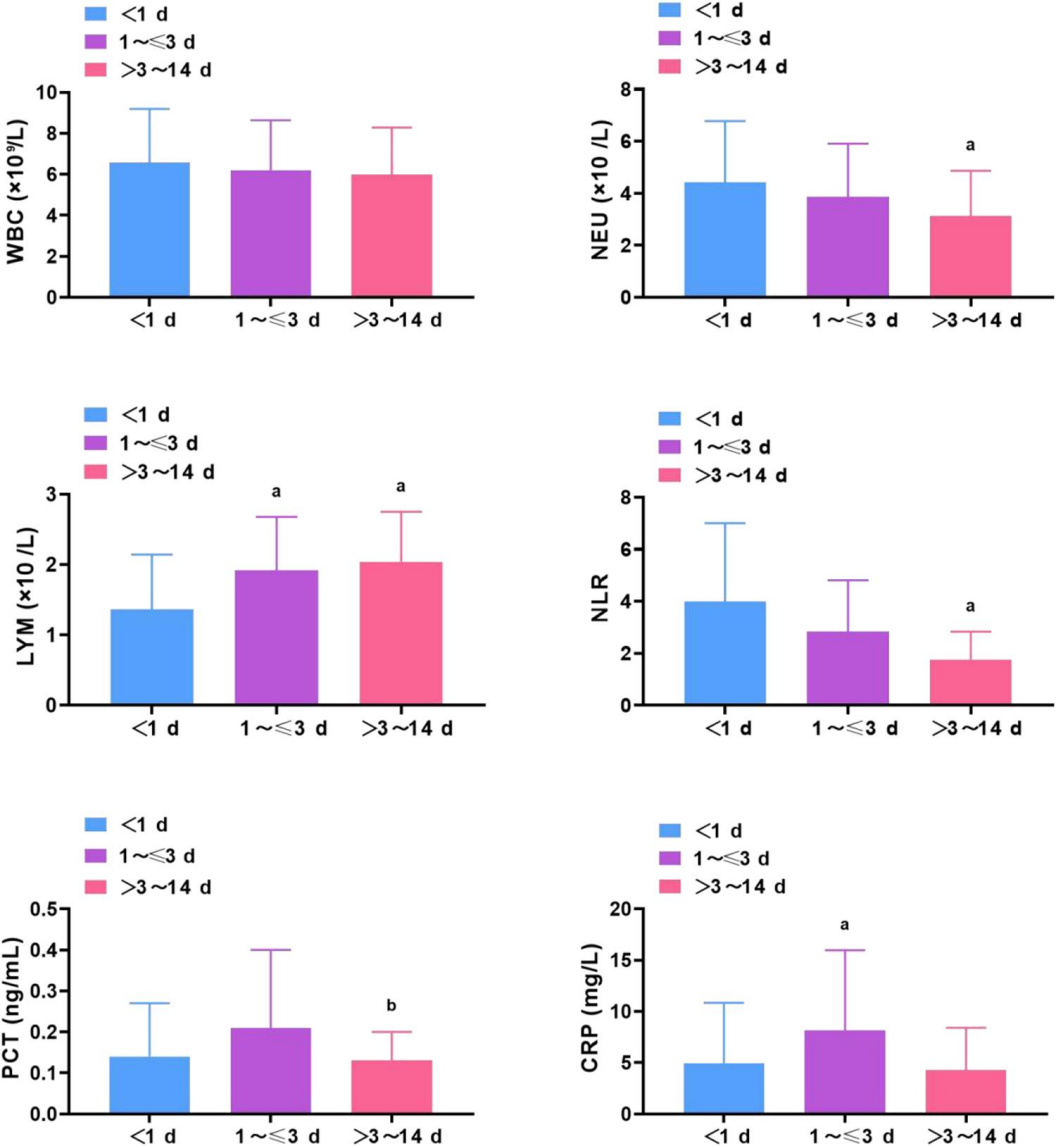
Changes in blood test indices over the disease course in children with influenza A. *P* values for pairwise comparisons were adjusted using Bonferroni correction. ^a^ indicates adjusted *P* < 0.05 compared with the <1 d stage; ^b^ indicates adjusted *P* < 0.05 compared with the >1–≤3 d stage.

With disease progression, hematological indices showed different trends. WBC showed a mild downward trend, but the difference was not statistically significant (*P* > 0.05). NEU gradually decreased and was lower in the >3–14 d stage than in the <1 d stage (*P* < 0.05). LYM gradually increased and was higher in both the 1–≤3 d and >3–14 d stages than in the <1 d stage (*P* < 0.05). NLR gradually decreased over the disease course and was lower in the >3–14 d stage than in the <1 d stage (*P* < 0.05). Regarding inflammation-related indicators, PCT and CRP showed an increasing trend in the 1–≤3 d stage and then decreased in the >3–14 d stage. Among them, PCT in the >3–14 d stage was lower than that in the 1–≤3 d stage (*P* < 0.05), and CRP in the 1–≤3 d stage was higher than that in the <1 d stage (*P* < 0.05). See Table 5 and Figure 2.

**Table 5:** Changes in blood test indices over the disease course in children with influenza A.

Item	<1 d (*n* = 100)	>1–≤3 d (*n* = 28)	>3–14 d (*n* = 15)
WBC (×10^9^/L)	6.58 ± 2.61	6.21 ± 2.43	5.98 ± 2.30
NEU (×10^9^/L)	4.42 ± 2.36	3.86 ± 2.05	3.12 ± 1.74^a^
LYM (×10^9^/L)	1.36 ± 0.78	1.92 ± 0.76^a^	2.04 ± 0.71^a^
NLR	3.98 ± 3.02	2.84 ± 1.97	1.76 ± 1.08^a^
PCT (ng/mL)	0.14 ± 0.13	0.21 ± 0.19	0.13 ± 0.07^b^
CRP (mg/L)	4.96 ± 5.88	8.14 ± 7.83^a^	4.28 ± 4.12

*P* values for pairwise comparisons were adjusted using Bonferroni correction.

a indicates adjusted *P* < 0.05 compared with the <1 d stage;.

b indicates adjusted *P* < 0.05 compared with the >1–≤3 d stage.

### 3.4 Tongue manifestation characteristics

Before consensus discussion, inter-rater reliability for tongue manifestation interpretation was substantial, with Cohen's kappa values of 0.84 for tongue color, 0.78 for coating texture, and 0.81 for coating color. During interpretation, age-related physiological tongue features were distinguished from disease-associated changes. In younger children, pale-red tongue or thin white coating was not regarded as a pathological heat or dampness sign unless supported by concurrent symptoms, disease-duration progression, or laboratory changes.

After applying these criteria, red tongue remained the predominant finding, observed in 134 cases (93.71%). Across disease-duration stages, the proportion of red tongue increased from 92.00% in the <1 d stage to 100% in the >3–14 d stage. Thick coating was observed in 91 cases (63.64%), and its proportion also increased with prolonged disease duration. White coating was the most common coating color, observed in 89 cases (62.24%); however, the proportion of yellow coating increased from 30.00% in the <1 d stage to 60.00% in the >3–14 d stage, suggesting progressive intensification of heat-related manifestations. See Table 6.

**Table 6:** Changes in tongue manifestations in children with influenza A.

Tongue manifestation	<1 d (*n* = 100)	>1–≤3 d (*n* = 28)	>3–14 d (*n* = 15)	Total (*n* = 143)
Tongue color	–	–	–	–
Pale red	6 (6.00%)	1 (3.57%)	0 (0%)	7 (4.90%)
Red	92 (92.00%)	27 (96.43%)	15 (100%)	134 (93.71%)
Dark purple	2 (2.00%)	0 (0%)	0 (0%)	2 (1.40%)
Coating texture	–	–	–	–
Thin coating	38 (38.00%)	6 (21.43%)	3 (20.00%)	47 (32.87%)
Thick coating	60 (60.00%)	20 (71.43%)	11 (73.33%)	91 (63.64%)
Greasy coating	2 (2.00%)	2 (7.14%)	1 (6.67%)	5 (3.50%)
Peeled coating	0 (0%)	0 (0%)	0 (0%)	0 (0%)
Coating color	–	–	–	–
White coating	70 (70.00%)	13 (46.43%)	6 (40.00%)	89 (62.24%)
Yellow coating	30 (30.00%)	15 (53.57%)	9 (60.00%)	54 (37.76%)

### 3.5 TCM syndrome differentiation

Among the 143 children, the TCM syndrome types ranked from high to low by number of cases were: heat-toxin attacking the lung syndrome (50 cases, 34.97%), wind-heat invading the exterior syndrome (47 cases, 32.87%), wind-cold constraining the exterior syndrome (29 cases, 20.28%), and dampness obstructing the defensive qi syndrome (17 cases, 11.89%). Stratified by sex, wind-heat invading the exterior syndrome was slightly more common in males (35.90%), while heat-toxin attacking the lung syndrome was slightly more common in females (38.46%). Stratified by age, wind-heat invading the exterior syndrome accounted for a higher proportion in children aged 0–≤5 years and >5–≤10 years, at 35.71% and 35.94%, respectively; children aged >10–16 years were mainly characterized by heat-toxin attacking the lung syndrome, accounting for 56.52%. Stratified by disease duration, the ≤1 d stage had a slightly higher proportion of wind-heat invading the exterior syndrome (33.00%); whereas the >3–14 d stage had the highest proportion of heat-toxin attacking the lung syndrome (53.33%), suggesting that as the disease course progresses, syndrome types tend to evolve from exterior to interior and from the defensive level to the qi level. See Table 7.

**Table 7:** TCM syndrome differentiation in children with influenza A.

Item	No. of cases (*n* = 143)	Heat-toxin attacking the lung syndrome	Wind-heat invading the exterior syndrome	Wind-cold constraining the exteriorsyndrome	Dampness obstructing the defensive qi syndrome
Sex	–	–	–	–	–
Male	78 (54.55%)	25 (32.05%)	28 (35.90%)	15 (19.23%)	10 (12.82%)
Female	65 (45.45%)	25 (38.46%)	19 (29.23%)	14 (21.54%)	7 (10.77%)
Age (years)	–	–	–	–	–
0–≤5	56 (39.16%)	17 (30.36%)	20 (35.71%)	12 (21.43%)	7 (12.50%)
>5–≤10	64 (44.76%)	20 (31.25%)	23 (35.94%)	14 (21.88%)	7 (10.94%)
>10–16	23 (16.08%)	13 (56.52%)	4 (17.39%)	3 (13.04%)	3 (13.04%)
Disease	–	–	–	–	–
≤1	100 (69.93%)	30 (30.00%)	33 (33.00%)	22 (22.00%)	15 (15.00%)
>1–≤3	28 (19.58%)	12 (42.86%)	9 (32.14%)	5 (17.86%)	2 (7.14%)
>3–14	15 (10.49%)	8 (53.33%)	5 (33.33%)	2 (13.33%)	0 (0%)
Total	143 (100.00%)	50 (34.97%)	47 (32.87%)	29 (20.28%)	17 (11.89%)

## 4 Discussion

This study systematically analyzed the clinical manifestations, dynamic changes in hematological indices, tongue manifestations, and syndrome differentiation patterns of children with influenza A in spring, depicting the disease characteristics and evolutionary trajectory of pediatric spring influenza A from multiple dimensions. Overall, the results showed features of abrupt onset, prominent heat signs, a tendency to be accompanied by gastrointestinal dysfunction, and relatively rapid syndrome transformation. This pattern is consistent with the immune-inflammatory response mechanisms of influenza virus infection and also aligns with the TCM understanding of warm diseases progressing “from the exterior to the interior, and from the defensive level to the qi level”. From the perspective of epidemiological characteristics and baseline data, the participants in this study were mainly school-age children, and most sought medical care within 1 d of onset. School-age children live in dense social environments with frequent contact opportunities, posing a high risk of droplet transmission (22). In addition, although the pediatric immune system has reached a certain level of maturity, specific immune memory against influenza virus remains imperfect, making children prone to clustered outbreaks during seasonal transitions (23). The fact that most children sought medical care at an early stage suggests, on the one hand, that spring influenza symptoms develop rapidly, and on the other hand, that the sample in this study more closely reflects the true acute-phase status of the disease; therefore, its depiction of early clinical and laboratory features has certain representativeness.

In terms of symptom distribution, fever remained the core manifestation and was mainly moderate to high fever. After influenza virus infection, interferons and various inflammatory mediators are induced and act on the hypothalamic thermoregulatory center, leading to elevated body temperature. Children have vigorous metabolism and active immune responses; therefore, the proportion of high fever is more prominent than in adults (24). In this study, the incidence of respiratory symptoms was also high, with cough accounting for more than 80%, suggesting that the virus primarily invades the respiratory epithelium, causing mucosal hyperemia and edema and increased secretions. Notably, the proportion of gastrointestinal symptoms was also high in this study, especially constipation, with an incidence exceeding 60%, and it became an important component of common symptom combinations. Previous studies (25, 26) have mostly focused on vomiting and diarrhea in children with influenza, while systematic statistics on constipation have been relatively scarce. The high frequency of constipation in this study should not be interpreted as evidence of a direct viral effect on the intestine. It may be more reasonably understood as a secondary response related to fever-induced fluid loss, reduced oral intake, decreased activity, inflammatory stress, or sympathetic activation, all of which may slow intestinal transit. From the TCM perspective, persistent heat may consume body fluids and impair the descending function of fu-qi, resulting in intestinal dryness and constipation (27). Therefore, constipation may serve as a useful accompanying sign when assessing heat accumulation or interior-excess tendency, but its mechanism needs further confirmation in prospective studies. The dynamic changes in hematological indices further revealed the immunological course of the disease. At the initial visit, LYM was markedly reduced, which is common in viral infections. Influenza virus can induce lymphocyte migration toward the site of infection, while inflammatory stress responses can promote lymphocyte apoptosis, leading to decreased peripheral blood counts (28). As the disease progressed, LYM gradually rebounded, whereas NEU and NLR gradually decreased, suggesting a transition from the acute inflammatory stress stage to the immune recovery stage. As a composite indicator reflecting the balance between inflammation and immunity, NLR is relatively elevated early in the disease and then declines, indicating a gradual weakening of inflammation-driven processes (29). PCT and CRP increased during the 1–3 d stage and then decreased, consistent with the pattern that the peak inflammatory response often occurs 2–3 d after onset (30). Although PCT typically does not rise significantly in viral infections, mild elevation may still occur in some children, possibly related to the intensity of individual inflammatory responses or local bacterial colonization. Importantly, dynamic trends provide greater clinical reference value than a single time point. A natural decline of inflammatory indices over the course often suggests entry into the recovery stage; persistent elevation warrants vigilance for secondary infection or disease progression. Therefore, the index changes observed in this study not only corroborate the immunological trajectory of influenza but also provide objective evidence for clinical monitoring and risk assessment. Recent pediatric TCM and integrated Chinese and Western medicine studies on influenza have mainly focused on treatment evaluation. For example, Qinxiang Qingjie oral solution has been evaluated in children with influenza and TCM-defined exterior-interior heat syndromes, while Xiao’er Fengre Qing oral liquid has been studied in pediatric influenza with wind-heat invading the defense syndrome (14, 16). In addition, a randomized controlled trial protocol has been designed to evaluate Reduning injection for pediatric influenza (15). Compared with these treatment-oriented studies, the present study focused on real-world clinical characteristics, hematological changes, tongue manifestations, and syndrome distribution across disease-duration stages. Our findings are generally consistent with previous syndrome-based pediatric influenza studies in that wind-heat and heat-related syndromes were common. More importantly, this study further suggests that tongue coating thickening, yellow coating, lymphocyte recovery, and changes in inflammatory indicators may provide complementary clues for understanding syndrome evolution during the disease course.

Changes in tongue manifestations corresponded internally with the above objective indices. The proportion of red tongue increased with longer disease duration, indicating gradually aggravated heat signs. The increasing proportions of thick coating and yellow coating suggested that the pathogenic factor progressed from the exterior to the interior or was accompanied by phlegm and dampness. In the early stage, white coating was more common, consistent with the characteristics of initial exogenous invasion when the pathogen remains in the lung–defensive level. With persistent fever or internal accumulation of pathogenic heat, the coating turns yellow and thickens, suggesting exuberant heat at the qi level or phlegm-heat obstructing the lung. Children tend to have a yang-predominant constitution; after being affected by wind-heat pathogens, they readily transform to heat. If heat pathogens are not cleared in time, body fluids are damaged and phlegm-dampness is generated internally, which may result in “ thick, greasy coating and yellow coating” (31). In this study, the proportion of yellow coating increased markedly in the later stage, consistent with the increased proportion of heat-toxin attacking the lung syndrome. The dynamic changes in tongue manifestations are not only changes in physical signs but also a direct reflection of pathomechanism transformation, providing important clues for dynamic TCM syndrome differentiation.

Syndrome differentiation results showed that wind-heat invading the exterior syndrome and heat-toxin attacking the lung syndrome were the main syndromes, with structural differences across age and disease duration. In younger children and school-age children, wind-heat invading the exterior syndrome predominated, suggesting that in most children the early stage remained at the lung–defensive level. In children aged >10–16 years, the proportion of heat-toxin attacking the lung syndrome increased markedly, possibly related to enhanced ability to express symptoms, more severe illness, or differences in visit timing. Stratification by disease duration showed that with time, the proportion of heat-toxin attacking the lung syndrome increased, indicating a trend of syndrome evolution from exterior to interior. This change was mutually corroborated by the increased proportion of yellow coating and the aggravation of thick coating in tongue manifestations. TCM theory holds that warm pathogens “ attack from above and first invade the lung”; if prolonged heat is not resolved and toxic heat accumulates internally, it transforms into exuberant qi-level heat or even lung heat obstruction (32, 33). The syndrome evolution observed in this study is highly consistent with this classical theory and also suggests that spring influenza A in children has the characteristic of “ rapid transmission and transformation.”

By integrating symptoms, indices, tongue manifestations, and syndrome types, a relatively complete disease-course evolution logic can be formed: in the early stage, fever and cough predominate, lymphocytes decrease, and red tongue with predominantly white coating is common, mostly corresponding to wind-heat invading the exterior syndrome; subsequently, inflammatory responses fluctuate, tongue coating gradually thickens and turns yellow, interior-excess manifestations such as constipation increase, and the syndrome transforms toward heat-toxin attacking the lung; in the later stage, inflammatory indices decline, immunity recovers, and tongue coating gradually recedes. This process is consistent with modern medical understanding of the immune response curve in viral infections and also fits the theoretical framework of warm diseases progressing from the defensive level to the qi level. The two explanatory pathways of Western medicine and TCM presented a relatively good mutual corroboration in this study, providing a practical basis for constructing an integrative understanding model. However, because treatment regimens before or after the first visit were not included as primary analytical variables, the observed syndrome distribution across disease-duration stages may have been influenced by antiviral therapy, antipyretic use, TCM treatment, or other symptomatic interventions. Therefore, these findings should be interpreted as disease-duration-related associations rather than a complete untreated natural disease course.

## 5 Clinical implications and limitations

The findings of this study may provide practical reference for the clinical assessment of pediatric influenza A in spring. Fever combined with cough remains the most suggestive symptom pattern, while constipation should also be noted as a frequent accompanying manifestation. However, constipation should not be regarded as a direct viral effect or an independent diagnostic feature; rather, it may reflect fever-related fluid loss, reduced intake, decreased activity, inflammatory stress, or heat-related intestinal dryness. In clinical practice, dynamic observation of symptoms, lymphocyte count, NLR, PCT, CRP, and tongue manifestations may help clinicians better understand disease-course changes and identify children who require closer monitoring. Tongue manifestations such as increasing redness, thick coating, and yellow coating may provide auxiliary clues for TCM syndrome differentiation, but they should be interpreted together with clinical symptoms and laboratory indicators rather than used alone.

This study also has several limitations. (1) This was a single-center retrospective study, and potential selection bias and information bias could not be completely avoided despite consecutive case screening and predefined eligibility criteria. (2) The sample size in the later stage (>3–14 d) was small, and the stability of some stage-based comparisons requires further verification. (3) Tongue interpretation was based on medical records and/or tongue photographs. Although unified categorical criteria, two-physician assessment, consensus discussion, and inter-rater reliability evaluation were used, standardized digital tongue image acquisition and quantitative colorimetric/texture analysis were not performed. (4) Treatment regimens and prognosis-related outcomes were not analyzed. Therefore, the observed syndrome evolution may have been influenced by antiviral therapy, antipyretic use, TCM treatment, or other symptomatic interventions, and the relationship among syndrome changes, treatment response, and clinical outcomes could not be determined.

Future primary research should adopt prospective, multicenter designs with larger sample sizes, especially for children with longer disease duration. Standardized digital tongue imaging, predefined syndrome differentiation procedures, and repeated assessments at fixed time points should be incorporated to improve objectivity and reproducibility. In addition, future studies should collect detailed treatment information and clinically meaningful outcomes, such as fever duration, symptom resolution time, complications, revisits, hospitalization, and treatment response, to clarify whether tongue manifestations and TCM syndrome patterns have predictive or decision-support value in pediatric influenza A.

## 6 Conclusion

In this single-center retrospective study, spring influenza A in children was mainly characterized by fever-dominant onset, frequent cough and constipation, early lymphopenia, dynamic changes in inflammatory indicators, and progressive changes in tongue manifestations across disease-duration stages. Red tongue and thick coating were common, and the proportion of yellow coating increased with longer disease duration. The main TCM syndrome types were wind-heat invading the exterior syndrome and heat-toxin attacking the lung syndrome, suggesting a possible disease-duration-related shift from exterior patterns toward intensified heat-related manifestations.

These findings may provide exploratory reference for integrated Chinese and Western medicine assessment of pediatric influenza A, particularly for dynamic observation of symptoms, hematological indicators, inflammatory markers, and tongue manifestations. However, the conclusions should be interpreted cautiously because this study was retrospective and single-center, the late-stage subgroup was small, treatment and outcome data were not analyzed, and standardized digital tongue imaging was not performed. Future prospective multicenter studies with standardized tongue image acquisition, repeated disease-course assessment, detailed treatment records, and clinically meaningful outcomes are needed to validate these findings and determine their value for clinical decision-making.

## Data Availability

The original contributions presented in the study are included in the article/Supplementary Material, further inquiries can be directed to the corresponding author/s.
